# The nature and consequences of coinfection in humans

**DOI:** 10.1016/j.jinf.2011.06.005

**Published:** 2011-09

**Authors:** Emily C. Griffiths, Amy B. Pedersen, Andy Fenton, Owen L. Petchey

**Affiliations:** a Department of Animal and Plant Sciences, Alfred Denny Building, Western Bank, University of Sheffield, Sheffield S10 2TN, UK; b Centre for Immunology, Infection and Evolution, Institutes of Evolutionary Biology, Immunology and Infection Research, Ashworth Labs, Kings Buildings, West Mains Road, University of Edinburgh, Edinburgh EH9 3JT, UK; c Institute of Integrative Biology, University of Liverpool, Liverpool L69 7ZB, UK; d Institute of Evolutionary Biology and Environmental Studies, University of Zürich, Winterthurerstrasse 190, Zürich CH-8057, Switzerland

**Keywords:** Coinfection, Concomitant disease, Host health, Integrated control, Parasite infracommunity, Pathogen abundance, Pathogen–host interactions, Pathogen diversity, Polymicrobial infection, Within-host parasite ecology

## Abstract

**Objective:**

Many fundamental patterns of coinfection (multi-species infections) are undescribed, including the relative frequency of coinfection by various pathogens, differences between single-species infections and coinfection, and the burden of coinfection on human health. We aimed to address the paucity of general knowledge on coinfection by systematically collating and analysing data from recent publications to understand the types of coinfection and their effects.

**Methods:**

From an electronic search to find all publications from 2009 on coinfection and its synonyms in humans we recorded data on i) coinfecting pathogens and their effect on ii) host health and iii) intensity of infection.

**Results:**

The most commonly reported coinfections differ from infections causing highest global mortality, with a notable lack of serious childhood infections in reported coinfections. We found that coinfection is generally reported to worsen human health (76% publications) and exacerbate infections (57% publications). Reported coinfections included all kinds of pathogens, but were most likely to contain bacteria.

**Conclusions:**

These results suggest differences between coinfected patients and those with single infections, with coinfection having serious health effects. There is a pressing need to quantify the tendency towards negative effects and to evaluate any sampling biases in the coverage of coinfection research.

## Introduction

The many pathogens that infect humans (e.g., viruses, bacteria, protozoa, fungal parasites, helminths) often co-occur within individuals.^1–5^ Helminth coinfections alone are thought to occur in over 800 million people,^6^ and are especially prevalent among the global poor.^7–9^ Other coinfections involve globally important diseases such as HIV,^10^ tuberculosis,^11^ malaria,^12^ hepatitis,^13^ leishmaniasis,^14^ and dengue fever.^15^ It seems likely, therefore, that the true prevalence of coinfection exceeds one sixth of the global population and often involves infectious diseases of pressing human concern.

Improved understanding of coinfection prevalence is greatly needed,^16^ partly because coinfecting pathogens can interact either directly with one another or indirectly via the host’s resources or immune system.^3^ Compared to infections of single pathogen species, these interactions within coinfected hosts can alter the transmission, clinical progression and control of multiple infectious diseases.^17–19^ Establishing the nature and consequences of coinfection requires integrated monitoring and research of different infectious diseases,^1^ but such data are rare.^9,20,21^

Reviews of coinfection have emphasised that coinfection requires further research, especially in humans,^2,3,20,22^ where coinfection outnumbers single infection in many communities^2,23^ and where helminth coinfections appear to worsen human health.^20^ Coinfection involves a range of pathogens and can have various effects on coinfected hosts.^3^ There are many individual studies concerning coinfection, but these use various approaches and are often narrowly focused. We aimed to gain a coherent picture of the nature and consequences of coinfection in humans. We surveyed the published literature for the occurrence of coinfecting pathogens and their effects on other infecting organisms and human health. We found that coinfections involve a huge variety of pathogens, and most studies report negative effects on human health. However, current coinfection research rarely focuses on pathogens with highest global mortality.

## Materials and methods

### Literature search

We searched the published literature for studies of coinfection (i.e. multi-species infections) in humans using the Advanced Search facility on the largest online citation database, Scopus (Elsevier Ltd.). Many disciplines study infectious diseases and various terms are used to describe coinfection. We therefore searched for coinfection, concomitant infection, multiple infection, concurrent infection, simultaneous infection, double infection, polymicrobial, polyparasitism, or multiple parasitism in the Title, Abstract, or Keywords of publications in the Life and Health Sciences before 2010. In June 2011 this search returned 12,963 results; an equivalent search on an alternative online citation database, Web of Science [Thomson Reuters], yielded similar trends in publications through time, but fewer results. Due to the large number of publications matching the search terms, we chose to focus on publications from 2009. Furthermore, publications concerning non-human hosts, non-infectious diseases or multiple genotypes of only one pathogen species were excluded.

For each publication we collected data on the identity of coinfecting pathogens, journal, study type and maximum number of pathogen species found per person. Study types included experiments treating each infection, observational studies, and reviews/meta-analyses. Observational studies were either case notes on particular patients, studies of patient groups, or epidemiological surveys among human communities.

Many publications reported the stated effect of one pathogen on the abundance of coinfecting pathogens (i.e. proxies for the intensity of infection, e.g. from measures of viral load, faecal egg counts, antibody response, bacterial cultures etc.) and/or host health (e.g. survival, recovery time, anaemia, liver fibrosis, immune cell counts). These effects of coinfection are relative to conditions observed under infections of single pathogen species. Where these effects were reported we recorded the pair of coinfecting pathogens involved, the quality of measurement (rated as low [e.g. anecdotal], adequate [e.g. correlation] and high [i.e. full reporting of appropriate statistical test supported by theoretical mechanisms]) and other data (see below). Data from review-type publications, case notes and from publications not mentioning the effects of coinfection (120 publications for pathogen abundance and 110 for host health) were excluded to avoid double counting, undue influence of individual cases and the inclusion of irrelevant publications. Reported effects based on low quality evidence (10 publications for pathogen abundance and 24 for host health) were also omitted.

### Analyses of the effects of coinfection

There was considerable heterogeneity in the reporting of the effects of coinfection, both in terms of the response variable and in terms of the quantitative measure given (e.g. odds ratios, adjusted odds ratios, *P*-values, hazards ratios, raw comparisons). Furthermore, many publications gave qualitative statements of effect direction. Among publications quantifying effect size, diverse measures were given across publications. We focused on the direction of reported effects (positive, negative and no-effect) to maximise the data available. Reported directions of the effects on both pathogen abundance and host health for each pair of coinfecting pathogens was coded +1 for positive effect, 0 for neutral, −1 for negative effects, and NA if no information about effect direction was given. The resulting dataset includes some repeated measures because some publications reported multiple pairs of coinfecting pathogens and some coinfections were reported in multiple publications. We created two independent datasets containing the mean effect direction (i) per publication and (ii) per coinfection to eliminate these sources of pseudoreplication. A negative mean implied a predominance of negative effects; a positive mean implied a dominance of positive effects. A mean close to 0 could result from either many neutral effects (whereby a pathogen consistently had no discernible effect) and/or equal numbers of positive and negative effects (whereby a pathogen had different, possibly context-dependent effects). In either case, there is no clear indication of these pathogens having a consistent effect on each other (or on host health), so we adopt the most conservative interpretation and assume there is no-effect. These means were converted into three categories: negative (−1 to −⅓), neutral (−1 to +⅓) and positive (+⅓ to +1). Chi-squared tests^24^ based on double log-likelihood values^25,26^ were done to establish whether totals in each category differed from those expected from two different null hypotheses (random and no-effect). The random null model was of equal proportions of positive, neutral and negative effects, while the no-effect null model was that coinfecting pathogens do not interact, allowing for a 5% error rate (hence 2.5% negative, 2.5% positive, and 95% neutral reported effects). This constitutes a recommended vote-counting method deriving continuous parameters analysed against confidence intervals (*α* = 0.05).^27^

Finally, we explored the potential influence of the missing data (NAs) on the effects of coinfection in the analysis (56 for pathogen abundance, 79 for host health). These values represent reported coinfections where the effect on either pathogen abundance or host health was not reported, despite the possibility that these coinfecting pathogens did interact with each other and/or influence host health. We therefore assessed how potential interactions from these unreported effects may alter the overall patterns of coinfection effects. To determine their potential impact on the estimated overall effects, NAs were assigned one of three values at random (+1, 0, −1). The mean effect was then calculated per publication or coinfection pair as before, and a grand mean taken across all publications or coinfection-pairs. The grand mean represents an estimate of overall effect of coinfection on either host health or pathogen abundance across either publications or coinfections, given a particular random assignment of −1, 0, +1 to NAs. Repeating this random assignment 1000 times produced a distribution of grand means.

### Comparison with WHO data

We examined whether recent coinfection research focuses on the pathogens causing the highest global mortality. We obtained global totals for the number of deaths (both sexes, all ages) in 2009 under every category of infection collated by the World Health Organisation (obtained from the Global Burden of Disease section of the Global Health Observatory website).^28^ We compared the ten categories causing most global deaths in 2009 with total reports of coinfection involving these infections. Comparing the top ten infection categories by mortality with their morbidity measures (DALYs) yielded similar trends, so we present only data from the mortality comparison.

## Results

### Overall trends in coinfection publications

**Figure 1:**
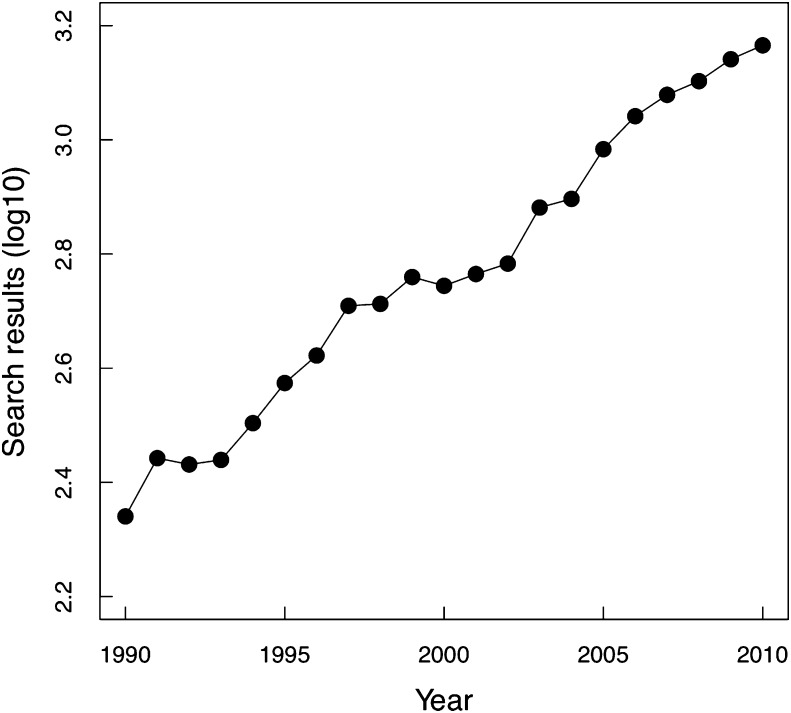
Annual coinfection publications (log_10_) from initial Scopus search. See the Methods section for search criteria.

Hundreds of publications on coinfection are published annually and have increased from 219 publications in the first year of search results to 1464 publications in 2009 (Fig. 1). This increase includes studies of both human and non-human hosts. Of the 1464 publications retrieved for 2009, 309 reported multiple pathogen species coinfecting humans. Publications came from 192 journals, with most (136 of 192 journals, 70.8%) publishing a single coinfection article in 2009.

The majority of relevant publications from 2009 were observational studies (234 of 309, 75.0%), of which 159 (67.9%) involved patient groups, 60 (25.6%) were case notes and 18 (7.7%) surveyed a population. Three observational studies (1.3%) analysed death records. Seventy publications (22.4%) were reviews or metaanalyses. Five publications (1.6%) were experimental, whereby treatment and controls were applied to both singly infected and coinfected groups. A majority of the relevant publications concerned coinfections by two pathogen species (249 of 309, 80.5%), but more pathogen species per individual were occasionally reported; the mean number of pathogens was 2.4 and a maximum of 13 pathogens was reported twice in a venous leg ulcer^29^ and a periodontal infection.^30^

### Reported coinfecting pathogens

A total of 270 pathogen taxa were reported in coinfection publications from 2009, across 1265 reports of coinfections comprising 933 different pairs of coinfecting pathogen taxa. All pathogen types (viruses, bacteria, protozoa, fungal parasites, helminths) were reported in coinfections; the most common pathogen group was bacteria (Table 1). In terms of specific pairs of reported coinfecting pathogens there was high diversity, but HIV and hepatitis viruses featured relatively highly (Table 1).

### Effects of coinfection on pathogen abundance and human health

Effects of coinfection on pathogen abundance and host health were sampled across 173 suitable publications according to pathogen abundance and host health. These publications covered 827 coinfecting pairs of pathogens, involving 183 pathogen species. Among these coinfections, 203 (24.5%) measured the size or direction of effects on pathogen abundance and 191 (23.1%) measured the size or direction of effects on host health. The remainder of coinfections had no reports of the effects of coinfection in suitable publications.

**Figure 2:**
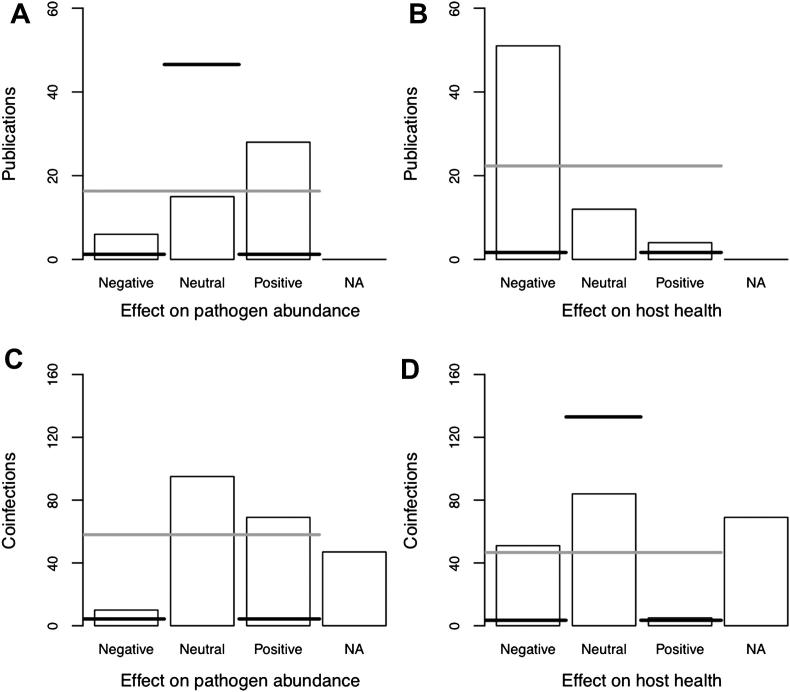
Direction of reported effects of coinfection on the abundance of infecting pathogens and host health averaged across publications and coinfections published in 2009. Horizontal lines indicate expected values of null hypotheses (black = no-effect, grey = random).

Overall, positive effects of coinfection on pathogen abundance were the most common reported across publications (6 negative, 15 neutral, 28 positive reports across 49 publications; Fig. 2A). Among specific pairs of coinfecting pathogens neutral effects exceeded positive effects (10 negative, 95 neutral, 69 positive across 174 unique pathogen pairs; Fig. 2C). In both cases these patterns were strongly significantly different from both the random null model (grey line on Fig. 2, by publication [*X*^2^ = 15.6, *d*.*f*. = 2, *P* < 0.001] and by coinfection [*X*^2^ = 82.6, *d*.*f*. = 2, *P* < 0.001]) and from the no-effect null model (black line on Fig. 2, by publication [*X*^2^ = 160.3, *d*.*f*. = 2, *P* < 0.001] and by coinfection [*X*^2^ = 292.8, *d*.*f*. = 2, *P* < 0.001]).

Regarding the impact of coinfection on host health, there was a much greater number of negative effects reported in publications than either positive, neutral or NA categories (51 negative, 12 neutral, 4 positive across 67 publications; Fig. 2B). When data were aggregated by specific pathogen pairs the neutral effects exceed the negative effects (51 negative, 84 neutral, 5 positive across 140 unique pathogen pairs; Fig. 2D). In both cases these patterns were significantly different from both the random null model (grey line, by publication [*X*^2^ = 55.6, *d*.*f*. = 2, *P* < 0.001, Fig. 2B] and by coinfection [*X*^2^ = 85.5, *d*.*f*. = 2, *P* < 0.001, Fig. 2D]) and from the no-effect null model (black line, by publication [*X*^2^ = 315.4, *d*.*f*. = 2, *P* < 0.001, Fig. 2A] and by coinfection [*X*^2^ = 199.6, *d*.*f*. = 2, *P* < 0.001, Fig. 2C]).

**Figure 3:**
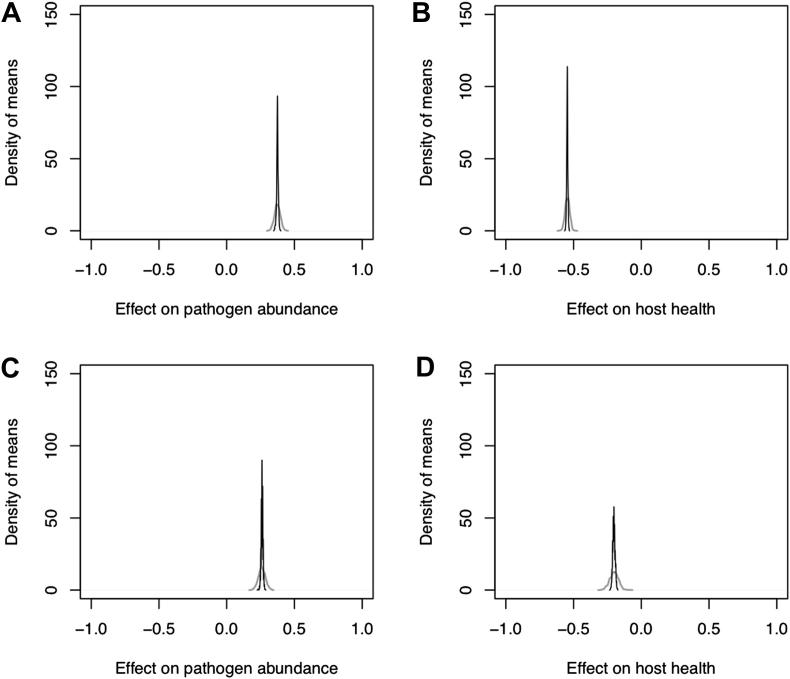
Distribution of grand mean effects of coinfection including simulations of missing values according to the random (grey line) and no-effect (black line) null models. Lines generated by a Gaussian kernel estimator (smoothing bandwidths: random = 5.1 × 10^−3^, no-effect = 1.2 × 10^−3^).

It is unlikely that these patterns of the effects of coinfection would be changed by knowledge of the unreported effects (the NAs in Fig. 2). Even after NA values were assigned predominantly to the neutral category (i.e. under the no-effect null model), the distribution of the grand mean effect was positive for the effects on pathogen abundance (Fig. 3A and C), and negative for effects on host health (Fig. 3B and D). None of the distributions of grand means overlapped zero (Fig. 3).

### Do coinfection studies focus on the most important infectious diseases?

**Figure 4:**
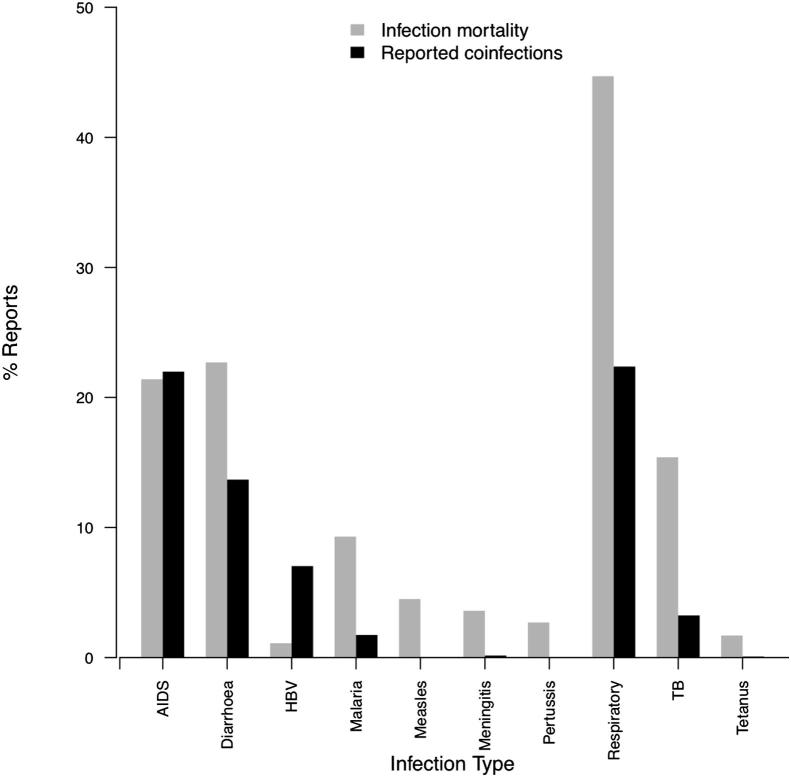
Top ten infections from global mortality data (28) (grey bars), compared with percentage of times the infections were reported in coinfections in 2009 publications (black bars).

We found notable differences between the most commonly reported coinfecting pathogens and the infections causing the greatest global health burden (Fig. 4). The largest infectious causes of mortality are respiratory infections, causing 44.7% of these deaths with the next greatest causes, diarrhoea and HIV/AIDS, causing half as many deaths. Other important infections by global mortality are tuberculosis, malaria and childhood infections (measles, meningitis, whooping cough and tetanus). The tenth biggest infectious cause of mortality worldwide, HBV, is the only hepatitis virus featuring in the top ten infectious causes of mortality, causing 1.1% of infectious disease deaths. In comparison, hepatitis viruses featured in one fifth of reported coinfections (286 of 1265, 22.6%). The top ten pathogen species reported in coinfections were HIV (in 266 [21.9%] of 1265 coinfections), HCV (11.4%), HBV (7.04%), *Staphylococcus aureus* (4.58%), *Escherichia coli* (4.43%), *Pseudomonas aeruginosa* (3.72%), *Mycobacterium tuberculosis* (5.9%), HPV (3.16%), unidentified *Streptococcus* spp. (3.00%), and unidentified *Staphylococcus* spp. (3.00%). Some of the most common reported coinfecting pathogens (HCV, *Staphylococcus*, HPV, and *Streptococcus*) contribute relatively little to global infection mortality. Perhaps surprisingly, four of the most important infectious causes of mortality (all of them childhood infections) received very few or no reports of coinfection in 2009 publications.

## Discussion

Interest in coinfection has increased in recent years, with publications on human coinfection involving hundreds of pathogen taxa across all major pathogen groups. Recent publications tend to show that negative effects of coinfection on human health are more frequent than no-effect or positive effects. However, the most commonly reported coinfecting pathogens differ from those infections causing highest global mortality. These results raise questions concerning the occurrence and study of coinfection in humans and their implications for effective infectious disease management.

The overall consequence of reported coinfections was poorer host health and enhanced pathogen abundance, compared with single infections. This is strongly supported by significant statistical differences in the reported direction of effects (*P* < 0.001) from expectations of either no-effect or of random distributions, and by the robustness of these trends in the face of missing values and by diversity in the types of publications in which these coinfections were reported. Moreover the tendency for positive effects on pathogen abundance corroborates the negative effects on host health because larger infections are a mechanism by which disease can be exacerbated. The consistency of these detrimental coinfection effects across a wide range of pathogens suggests a general incidence of interactions between coinfections. The long-term effects among survivors of coinfections can be varied and in some cases severe, including blindness, chronic diarrhoea, chronic inflammation, carcinoma, immunosuppression, liver fibrosis, meningitis, renal failure, rheumatic fever, *etc*.^31^

The direction of reported coinfection effects could have at least two explanations. The first is that coinfection may be more likely in individuals of poor health, which in turn leads to poorer prognosis among coinfected cases. The relative paucity of experimental studies of coinfection in humans means sampling biases towards people of poorer health is possible, but impossible to account for in our analyses. The second explanation is that coinfecting pathogens interact synergistically with each other, for example via the host’s immune system, so that the presence of one enhances the abundance and/or virulence of the other. A clear example of this is HIV, which causes immunosuppression, increasing the likelihood of additional infections and occurred in two fifths of reported coinfections (Fig. 4).

Differences between reported coinfections and global mortality figures may also suggest important interactions between coinfecting pathogens. Coinfections that were more commonly reported than their relative contribution to global mortality may involve particular synergistic pathogen–pathogen interactions, such as among herpes viruses like CMV or HSV infection enhancing the risk of HPV coinfection.^32^ Conversely, infections that cause high mortality but had relatively few reports of coinfection could result from antagonistic interactions, reducing the likelihood of such coinfections occurring and being reported, like *P. aeruginosa* exoproduct limiting *S. aureus* colony formation.^33^ An alternative and possibly more likely explanation of the discrepancies between reported coinfections and global mortalities from infections could be greater funding availability (e.g. HIV/AIDS research), higher interests of virologists in coinfection and/or easier observations or more routine screening compared with other pathogens, for instance the greater difficulty of detecting intestinal helminths in coinfection research. The lack of coinfection publications reporting on major infectious causes of childhood mortality remains unexplained. While some publications do study childhood coinfection and find coinfection to be more common in children,^34^ current coinfection research does not include the infections that kill the most infants globally. Fewer than 1 in 20 publications reported coinfections involving helminths, despite hundreds of millions of helminth coinfections globally,^6^ which could arise from limited published research on helminthiases. To what extent disparities between global mortality data reflect actual epidemiology or biases in research attention remains to be established, in part hindered by current inadequacies in coinfection surveillance.

The disparity between infections that feature highly in global mortality statistics and those receiving most attention in published coinfection studies poses a challenge to infectious disease research. A general understanding of the effects of coinfection is important for appropriate control of infectious diseases.^4,7,8,35^ Poor or uncertain observational data regarding coinfection hinders efforts to improve health strategies for infectious disease in at-risk populations.^9^ For example, global infectious disease mortality data^28^ report only single causes of death, even if comorbidities were identified. If health statistics better represent coinfection, published coinfection research could be better evaluated. Moreover there is a lack of coherence in coinfection literature, with a variety of synonyms being used for the same phenomenon, which is multi-species infection (see the Methods for examples). The term polymicrobial, while commonplace, is restricted to coinfections involving microbes. Coinfection is a broader term encompassing all pathogen types including interactions between the same kinds of pathogens as well as cross-kingdom coinfections between, say, bacteria and helminths. Ultimately decisions over which term to prefer (if any) need to be made by a consensus of the diverse research communities concerned with this phenomenon. True patterns of coinfection remain unknown^21^ and our results suggest that it may be starkly different from existing data on important infectious diseases.

Overall recently published reports of coinfection in humans show coinfection to be detrimental to human health. Understanding the nature and consequences of coinfection is vital for accurate estimates of infectious disease burden. In particular, more holistic data on infectious diseases would help to quantify the size of the effects on coinfection on human health. Improved knowledge of the factors controlling an individual’s risk of coinfection, circumstances when coinfecting pathogens interact, and the mechanisms behind these pathogen–pathogen interactions, especially from experimental studies, will also aid the design and evaluation of infectious disease management programmes. To date, most disease control programs typically adopt a vertical approach to intervention, dealing with each pathogen infection in isolation. If coinfecting pathogens generally interact to worsen human health, as suggested here, control measures may need to be more integrated and specialist treatments developed for clinical cases of coinfection. Further research is needed to identify the role of predisposed risks to coinfection.

## Figures and Tables

**Table 1:** Number of reports of each type of pathogen and the five most reported pairs of coinfecting pathogens among 2009 coinfection publications.

Pathogen Type	Frequency (%)	Coinfecting pathogens	Frequency (%)
Bacteria	1351 (53.4)	HCV-HIV	82 (6.5)
Viruses	877 (34.7)	HBV-HIV	31 (2.4)
Protozoa	117 (4.6)	HBV-HCV	30 (2.4)
Helminths	78 (3.1)	HIV-*Mtb*	28 (2.2)
Fungi	81 (3.2)	HIV-HPV	27 (2.1)

HBV = Hepatitis B Virus, HCV = Hepatitis C Virus, HIV = Human Immunodeficiency Virus, *Mtb* = *Mycobacterium tuberculosis*, HPV = Human Papillomavirus.
